# Extracellular mitochondrial DNA activates complement and is associated with complement activation in patients with out-of-hospital cardiac arrest

**DOI:** 10.1038/s41598-025-24705-1

**Published:** 2025-11-20

**Authors:** Eline de Boer, Anne-Lise Strandmoe, Marina Sokolova, Trond M. Michelsen, Huy Q. Quach, Viktoriia Chaban, Espen R. Nakstad, Geir Ø. Andersen, May-Kristin S. Torp, Kåre-Olav Stensløkken, Tom E. Mollnes, Søren E. Pischke

**Affiliations:** 1 Institute of Clinical Medicine, University of Oslo, Oslo, Norway; 2 Department of Immunology, Oslo University Hospital, Oslo, Norway; 3 Department of Medical Biology, Department of Medical Sciences, University of Groningen, Groningen, the Netherlands; 4 Department of Obstetrics, Oslo University Hospital, Oslo, Norway; 5 General Internal Medicine, Mayo Clinic, Rochester, MN USA; 6 Department of Acute Medicine, Oslo University Hospital, Oslo, Norway; 7 Department of Cardiology, Oslo University Hospital, Oslo, Norway; 8 Institute of Basic Medical Sciences, University of Oslo, Oslo, Norway; 9 Research Laboratory, Nordland Hospital Bodø, Bodø, Norway; 10 Department of Anaesthesiology and Intensive Care, Division of Emergencies and Critical Care, Oslo University Hospital, Oslo, Norway; 11 Division of Emergencies and Critical Care, Department of Immunology, Oslo University Hospital, P. B. 4950, Nydalen, Oslo, 0424 Norway

**Keywords:** Acute inflammation, Complement cascade, Innate immunity, Translational research

## Abstract

Synthetic analogues of mitochondrial DNA (mtDNA) have been reported as potent complement system activators. This study investigated the impact of endogenous mtDNA on complement activation. mtDNA and nuclear DNA (nDNA) were extracted from human placental tissue and complement activation was determined using ELISA. When incubated in lepirudin-anticoagulated human blood and plasma, increasing concentrations of mtDNA, but not nDNA, resulted in dose- and time-dependent increases in C3bc, C3bBbP and soluble C5b-9 (sC5b-9). In a clinical context, mtDNA, nDNA, and complement levels of 55 resuscitated out-of-hospital cardiac arrest (OHCA) patients were determined using qPCR and ELISA. C3bc and sC5b-9 correlated significantly with mtDNA and nDNA in OHCA patients. We conclude that mtDNA, but not nDNA, triggers in vitro complement activation, which holds significance in a clinical context in OHCA patients. This novel mode of human complement activation might explain pathophysiological mechanisms of sterile inflammation and could be relevant for innovative therapeutic strategies.

**Supplementary Information:**

The online version contains supplementary material available at 10.1038/s41598-025-24705-1.

## Introduction

 Sterile systemic inflammatory responses are activated in acute conditions such as severe trauma and cardiac arrest, which all lead to ischemia reperfusion injury (IRI) and can result in multiorgan failure and death^1,2^. All molecular mediators involved in the pathogenesis of these proinflammatory responses remain yet to be identified^3^. Extracellular mitochondrial DNA (mtDNA) has attracted increasing attention as it has been recognized as a damage-associated molecular pattern (DAMP) capable of eliciting immune responses^4–6^. There is strong evidence that mtDNA plasma levels are significantly elevated in various critical illnesses^7^.

mtDNA has a bacterial ancestry and contains immunostimulatory hypomethylated cytosine-phosphate-guanine (CpG) motifs^8^. When released into the circulation under conditions of necrosis or tissue damage, mtDNA is thought to drive inflammation toward a pro-inflammatory state through the activation of several pattern recognition receptors (PRRs) such as toll-like receptor (TLR)-9, NOD-like receptor protein 3-inflammasome and cyclic GMP-AMP synthase stimulator of interferon gene^6,9^. Recently, we and others suggested a contributing role for the complement system, as synthetic analogs of mtDNA were shown to activate the complement cascade^10,11^. Yet, the effect of endogenous mtDNA on complement activation remains unclear.

The complement cascade is an important component of innate immunity which protects the host from invading pathogens and eliminates cellular debris through C3b opsonization and C5b-9 mediated cell lysis^12,13^. However, excessive release of DAMPs into the circulation may lead to inappropriate and dysregulated complement activation and could result in detrimental inflammatory responses. Systemic inflammation as a part of IRI following cardiac arrest is thought to play a potential role in anoxic brain injury^14^. Further, plasma levels of mtDNA have been correlated with sC5b-9 and adverse clinical outcomes in hospitalized COVID-19 patients^15^.

To elucidate the role of complement activation in response to mtDNA and within cardiac arrest-imposed inflammation, we aimed to investigate (1) the effect of mtDNA as compared to nDNA on the complement system in human whole blood and plasma, and (2) the relation between plasma levels of mtDNA and nDNA and complement products in out-of-hospital-cardiac arrest (OHCA) patients.

## Materials and methods

### Mitochondrial and nuclear DNA isolation

Placental tissues from five donors with healthy pregnancies were obtained under sterile conditions immediately after scheduled caesarean section in the operating theatre. Tissue was obtained through random sampling from all placental quadrants and stored at -80 °C until use. The frozen tissue was cut into small pieces of approximately 200 mg, washed twice with ice-cold sterile phosphate-buffered saline (PBS), and homogenized for 5 s at 3000 rpm (GentleMACS Dissociator, Miltenyi Biotec, Bergisch Gladbach, Germany) in pre-chilled isolation buffer from the Mitochondria Isolation Kit for Tissue (Thermo Fisher Scientific, Waltham, MA). Initial centrifugation (700x*g*, 20 min, 4 °C) separated mitochondria from nuclei, connective tissue fibers and whole cells. In addition to the washing step recommended by manufacturer´s instructions, two additional steps of washing with buffer C were performed after the 3000x*g* centrifugation to minimize nDNA carry-over. The crude nuclear pellet and the mitochondria pellet were washed twice with PBS and resuspended in 600 µl TE-Tween buffer. DNA extraction and purification were performed according to the protocol described by Dani *et al*.^16^. In short, DNA was purified by standard enzymatic digestion of proteins including proteinase K and RNase, and phenol-chloroform-isoamyl (PCI) DNA extraction. A control sample containing only PBS followed the same procedure at the same time as for mtDNA and nDNA extraction. Aliquots from each of the five donors were pooled.

### Quantitative real-time polymerase chain reaction

mtDNA and nDNA were quantified as copy number (copies/µl), as this more directly reflects ligand availability than DNA mass. mtDNA and nDNA in the samples were quantitated in duplicate by quantitative real-time polymerase chain reaction (qPCR). To calculate mtDNA copy numbers, human nicotinamide adenine dinucleotide dehydrogenase 1 (ND1) cDNA clone (SC101172, Origene Technologies, Rockville, MD) was used. To prevent overestimation of copy numbers, a heat-linearization step was applied on the supercoiled ND1 plasmid (15 min at 95 °C)^17^. To calculate nDNA copy numbers, human genomic DNA encoding for GAPDH (G3041, Promega, Madison, WI) was obtained. The standard curves were established by 10-fold serial dilutions of ND1 and GAPDH and analysed together with the samples. The DNA copy number calculator (http://cels.uri.edu/gsc/cndna.html; University of Rhode Island Genomics and Sequencing Center, Kingston, RI) was used to convert plasmid and genomic DNA concentrations into copy numbers. Each reaction contained a mixture of 2 µl DNA, 1 µl gene-specific primers (ND1:4351370 or GAPDH:4331182, Thermo Fisher Scientific), 10 µl TaqMan PCR Master Mix (444963, Thermo Fisher Scientific) and 7 µl nuclease-free water. qPCR was performed on a StepOnePlus Real-Time PCR machine from Applied Bioscience (Thermo Fisher Scientific) using the following conditions: denaturation step of 2 min at 50 °C and 2 min at 95 °C, followed by 40 cycles at 95 °C for 1 s and 60 °C for 20 s.

### Whole blood and plasma experiments

Human whole blood was obtained from healthy volunteers (*n* = 6) and collected into sterile polypropylene tubes (Nalgene NUNC, Roskilde, Denmark) containing 50 µg/ml lepirudin (Refludan, Pharmion, Copenhagen, Denmark). Plasma was obtained by centrifugation of whole blood (3000x*g* for 15 min at 4 °C). Aliquoted whole blood and plasma were diluted 1:4 in PBS and incubated with increasing, equivalent copy numbers of pooled mtDNA- and nDNA in a 37 °C shaking water bath for up to 1 h. Complement activation reactions were terminated by addition of 10 mM EDTA. Plasma was collected and stored at -70 °C until further analyses.

### Complement activation markers

Plasma levels of complement activation products, including C3bc, C3bBbP and fluid phase C5b-9 (sC5b-9), were assessed by standardized in-house developed enzyme-linked immunosorbent assays (ELISA), as described previously^18–21^. sC5b-9 data points from one donor were excluded due to technical issues.

### The Norwegian cardio-respiratory arrest study

EDTA plasma samples on admission from the Norwegian Cardio-Respiratory Arrest Study (NORCAST, NCT01239420) were used. Resuscitated adult OHCA patients were prospectively enrolled on admission to Oslo University Hospital Ullevål between 2010 and 2014. Detailed information regarding the patient population in NORCAST can be found elsewhere^22^. The markers of endothelial activation and damage soluble (s)Syndecan-1, soluble (s)E-selectin, soluble thrombomodulin (sTM), soluble vascular cell adhesion molecule 1 (sVCAM-1) as well as soluble CD14 were selected based on previous findings in the NORCAST cohort documented by Chaban *et al*.^23^, where these endothelial markers on day three were shown to increase after OHCA and to be associated with neurological outcome. The quantification of C3bc and sC5b-9 at admission as well as the endothelial markers and soluble (s)CD14 quantification after 72 h had been previously conducted by Chaban *et al*.^23^. In this sub study, we applied a stratified random sampling approach based on pre-defined exploratory complement activation categories (none [sC5b-9 < 0.7 CAU/ml]; mild [sC5b-9 > 0.7-2 CAU/ml]; moderate [sC5b-9 > 2–5 CAU/ml]; severe [sC5b-9 > 5 CAU/ml]) and neurological outcome obtained within six months after cardiac arrest (cerebral performance category (CPC) score, good outcome (1–2); bad outcome (3–5) yielding eight strata. Seven patients per stratum were randomly selected using a random number generator (Excel RAND function, Microsoft Corp., Redmond, USA), resulting in 56 patients; one was excluded due to insufficient plasma, to assure an equal representation of the NORCAST study cohort. Clinical characteristics of the study cohort are summarized in Table 1. DNA was isolated using the QIAamp DNAeasy Blood & Tissue Kit (Qiagen, Manchester, UK).

### Ethical statement

This study was designed and performed according to the ethical guidelines from the declaration of Helsinki. The Regional Committee for Medical and Health Research Ethics, South-East Norway (REK Sør-Øst) approved the use of placenta tissue (REK 2013/624), blood from healthy donors (REK 05/197), and the NORCAST study (REK 2010/1116a). Only participants aged 18 years or older (i.e., above the legal age of consent in Norway) were included in the studies described. Informed consent was obtained from all participants or their next of kin if unconscious at time-point of inclusion in the NORCAST study and from the participant if ability to give informed consent was regained within six months of hospitalization.

### Statistical analyses

Statistical analyses were performed with IBM SPSS Statistics for Macintosh version 28 (IBM Cooperation, Armonk, NY) and GraphPad Prism 9 (GraphPad Software, San Diego, CA). To minimize inter-plate variation, complement values were normalized by subtracting the control value, wherein PBS was used as a substitute for DNA. The generalized linear mixed model analysis was used to compare the degree of complement activation over time between the DNA and PBS-treated control group within each donor. The fixed intercept consisted of the variables time, group, and time-by-group, while the random intercept included the individual donor. One plasma donor was excluded from analyses due to a too small working volume of the cell-free PBS sample which was used as a reference value. Associations between complement activation products, mtDNA, nDNA, endothelial markers and clinical parameters were examined using Spearman´s rank-order correlation method. All tests were considered statistically significant if the 2-sided p value was < 0.05.

**Table 1:** Clinical characteristics.

	Total (*n* = 55)
Age (years)	62 (55–70)
Sex	
Female	4 (7%)
Male	37 (67%)
Cause of cardiac arrest	
Acute myocardial infarction	21 (38%)
Chronic coronary disease	17 (31%)
Arrhythmic ventricular fibrillation	6 (11%)
Hypoxia induced cardiac arrest	7 (13%)
Other causes	1 (2%)
Creatinine µmol/l	111 (88–134)
Urea mmol/l	7 (6–8)
SOFA score	11(10–12)
Time to ROSC (min)	25 (14–33)
Survivors	30 (55%)
Deaths	
Cardiac cause	5 (9%)
Cerebral cause	13 (24%)
Other cause	3 (6%)
Unknown	1 (2%)

Data presented as median (1st -3rd quartile) or n (%). SOFA; Sequential Organ Failure Assessment, Time-to-ROSC; time to return of spontaneous circulation, min; minutes.

## Results

### Production and relative yield of nuclear to mitochondrial DNA

**Fig. 1:**
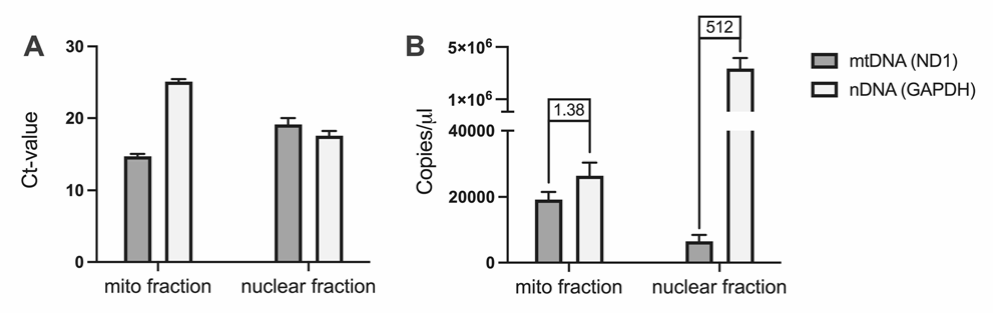
Quantity of mitochondrial (mtDNA) and nuclear DNA (nDNA) in the mitochondrial and nuclear fraction. The absolute concentrations of ND1 (mtDNA) and GAPDH (nDNA) in each of the fractions were derived by implementing their Ct-values (A) on a standard curve of plasmid (ND1) and human genomic DNA. The pooled mitochondrial fraction contained 1.38 times more nuclear than mitochondrial DNA and the pooled nuclear fraction contained 512 times more nuclear than mitochondrial DNA (B). Data are presented as mean ± SEM.

mtDNA and nDNA was purified from human placental tissue and the Ct-values of ND1 (mtDNA) and GAPDH (nDNA) were implemented on standard curves of a plasmid (ND1) and human genomic DNA to determine the absolute quantity of mtDNA and nDNA in each of the fractions (Fig. 1A). DNA extraction from the isolated mitochondria resulted in an average mtDNA-yield of 19,200 copies/µl and an nDNA-yield of 26,500 copies/µl. DNA extraction from the crude nuclei had an average mtDNA yield of 6,500 copies/µl and an nDNA yield of 3,345,700 copies/µl. The ratio of nDNA: mtDNA for the mitochondrial fraction was 1.38 and for the corresponding nuclear fraction 512 (Fig. 1B).

### Complement was activated by MtDNA but not by nDNA, in whole blood and plasma

**Fig. 2:**
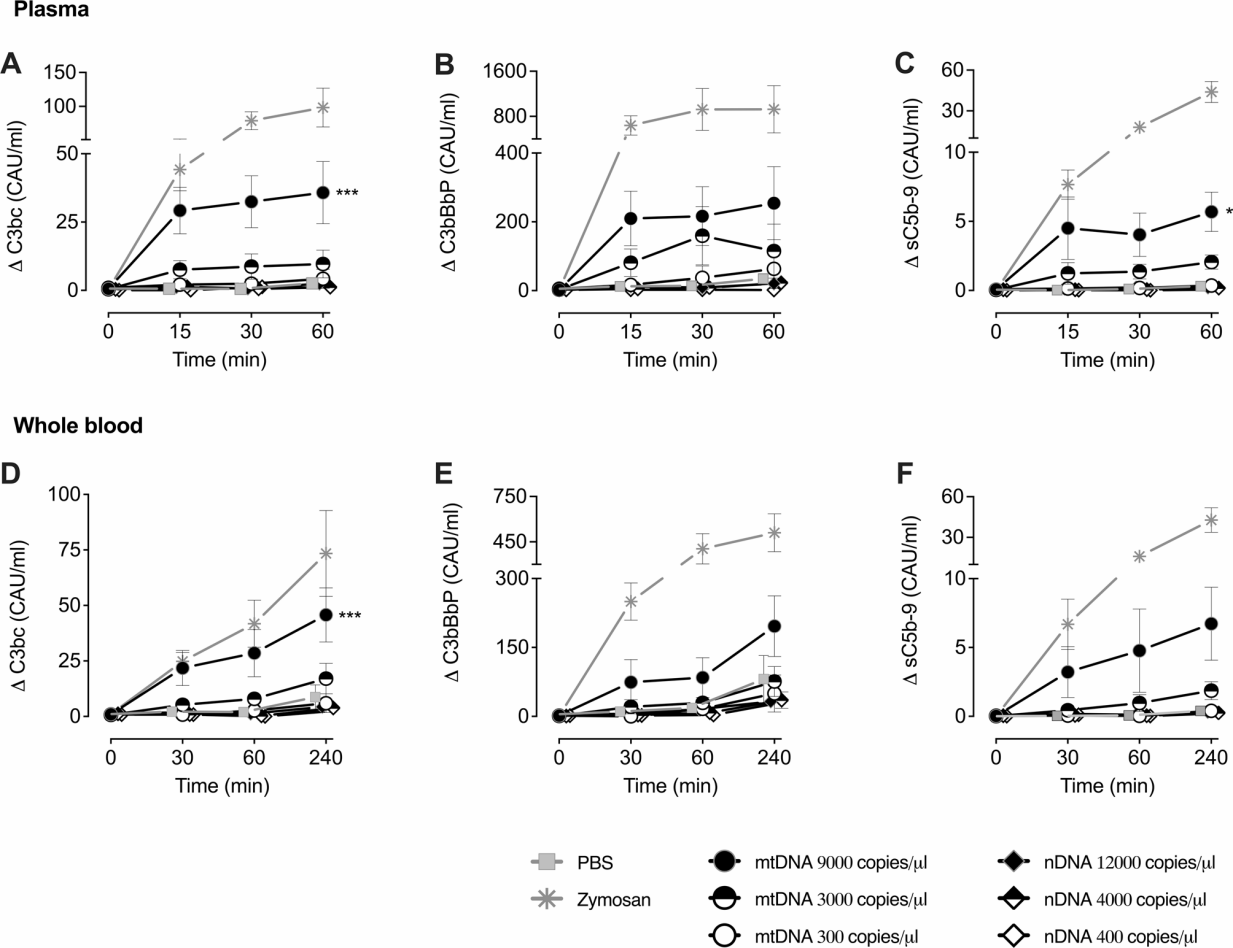
A mtDNA induced complement activation in whole blood and plasma. Human whole blood (A-C) or plasma (D-F) was incubated with increasing concentrations of a mtDNA-enriched fraction and nDNA-fraction. C3bc (A, D), C3bBbP (B, E), and sC5b-9 (C, F) increased in a dose- and time-dependent manner of the mtDNA-enriched fraction and zymosan (100 µg/ml) serving as positive control. Thus, significant increases in the presence of mtDNA and zymosan were observed for C3bc (A, D) and sC5b-9 (C), whereas nDNA had no effect on complement activation. Data are presented as mean ± SEM. sC5b-9 data points from one donor were excluded (due to technical issues); A,D (*n* = 6), B, E (*n* = 6), and C, F (*n* = 5). General mixed model analyses. * = *p* < 0.05, *** = *p* < 0.001. CAU; complement arbitrary units.

The incubation of mtDNA-enriched fraction with either human whole blood or plasma led to a dose- and time-dependent increase of complement activation products, including C3bc, C3bBbP and sC5b-9 (Fig. 2). The highest concentration of 9000 copies/µl mtDNA led to significantly higher C3bc and sC5b-9 in plasma (*p* < 0.001; *p* = 0.031, respectively) and in whole blood (*p* < 0.001; *p* = 0.055, respectively) compared with the negative control. Meanwhile, nDNA incubation was not significantly different from the negative control (Fig. 2). Normalization of TCC and C3bc responses in plasma and whole blood to total DNA confirmed that complement activation was driven by mtDNA (data not shown).

### Associations between plasma MtDNA and nDNA, complement levels, clinical parameters, and damage biomarkers in OHCA patients

**Fig. 3:**
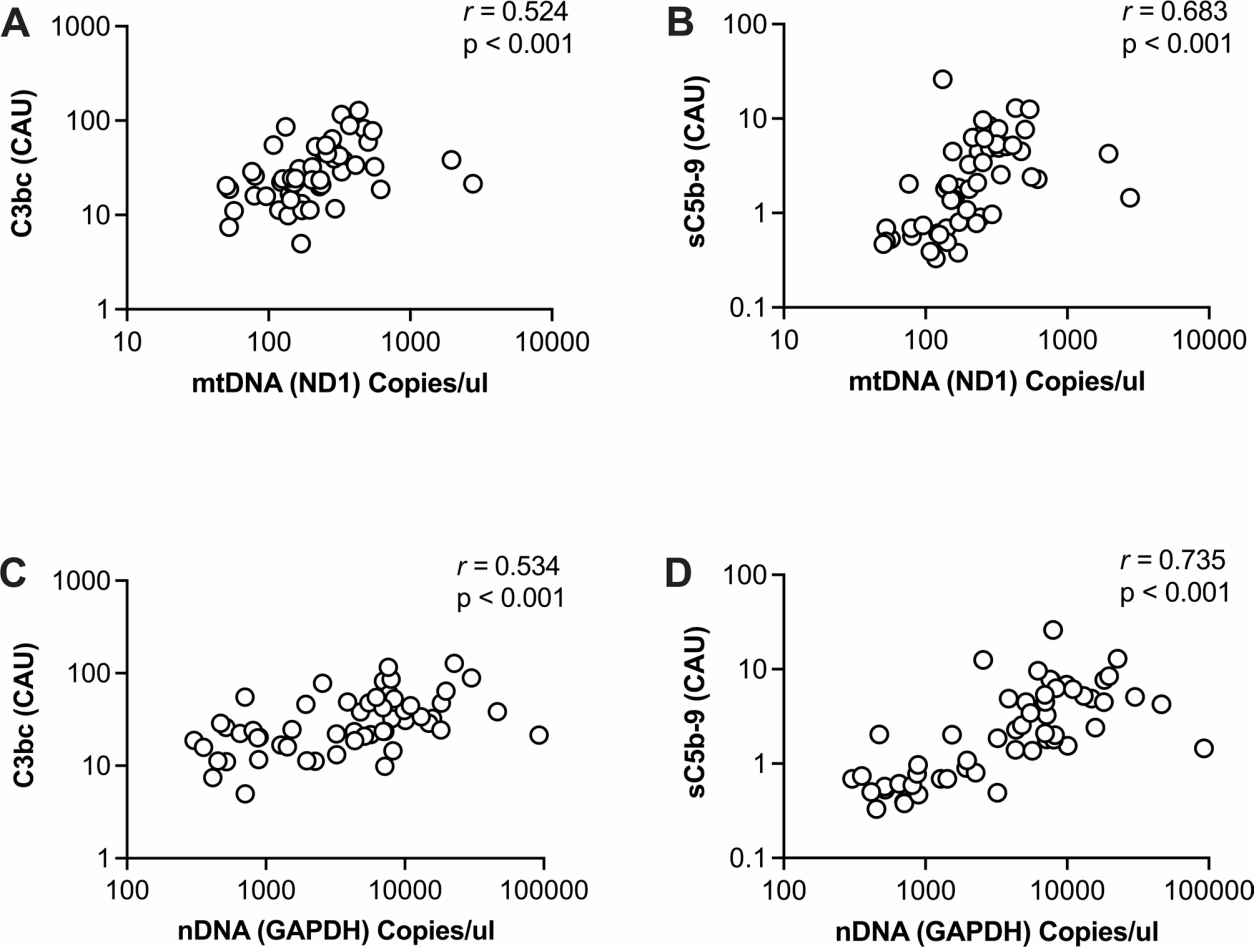
Correlation among circulating mtDNA, nDNA and complement proteins. Significant positive correlations were observed for mtDNA and nDNA between plasma concentrations of C3bc (A, C) and sC5b-9 (B, D) in out-of-hospital cardiac arrest patients. Spearman´s correlation. CAU; complement arbitrary units.

Samples from OHCA patients showed a mean mtDNA concentration of 307 copies/µl. Plasma levels of mtDNA and nDNA were significantly and strongly correlated to each other (*r* = 0.683, *p* < 0.001), moderately correlated with the degree of C3bc (*r* = 0.524, *p* < 0.001; *r* = 0.534, *p* < 0.001, respectively), and strongly with sC5b-9 (*r* = 0.683, *p* < 0.001; *r* = 0.735, *p* < 0.001, respectively) in OHCA patients (Fig. 3). Weak correlations were observed between mtDNA concentrations and the clinical variables SOFA-score and time-to-return-of-spontaneous circulation (Time-to-ROSC) (*r* = 0.293, *p* = 0.037; *r* = 0.321, *p* = 0.036, respectively) (Table 2). Among the cellular markers, only sCD14, a co-receptor of TLRs, was weakly positively correlated with nDNA levels in OHCA patients. Regression analyses of mtDNA plasma concentrations with clinical outcomes did not reveal statistically significant associations (Supplemental Table 1).

**Table 2:** Correlations between DNA plasma levels, scores at admission and plasma damage biomarkers in the NORCAST study.

	Correlation with mtDNA		Correlation with nDNA	
	*r*	p-value	*r*	p-value
Scores at admission				
SOFA-score	0.293	**0.037**	0.183	0.199
Time-to-ROSC	0.321	**0.036**	0.290	0.060
Plasma levels				
sCD14	0.271	0.086	0.355	**0.023**
Syndecan-1	-0.038	0.812	-0.168	0.294
E-selectin	0.096	0.551	0.189	0.237
VCAM-1	0.023	0.885	0.063	0.698
Thrombomodulin	-0.038	0.814	-0.047	0.769

mtDNA, mitochondrial DNA; nDNA, nuclear DNA; r, correlation coefficient; SOFA, sequential organ failure assessment; Time-to-ROSC, time-to-return-of-spontaneous circulation, VCAM, vascular cell adhesion molecule.

## Discussion

**Fig. 4:**
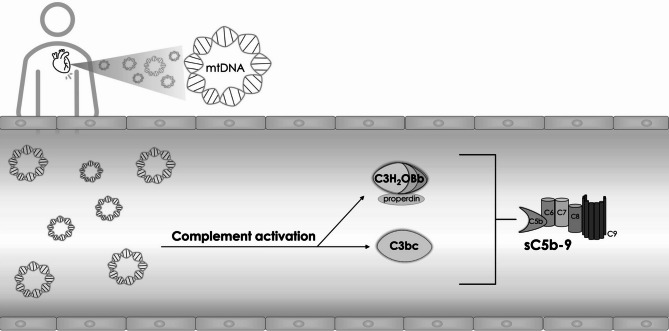
Representation of the proposed interaction between mtDNA and complement activation in vitro and in out-of-hospital cardiac arrest patients.

In this study, we purified mtDNA and nDNA from human placental tissue and achieved a high relative yield of mtDNA in the mitochondrial fraction. mtDNA, but not nDNA, activated complement pathways in human whole blood and plasma in a dose- and time-dependent manner. This complement activation was further correlated with levels of circulating mtDNA and nDNA in patients who experienced out-of-hospital cardiac arrest (OHCA), highlighting a significant association with complement activation markers, particularly C3bc and sC5b-9 (Fig. 4).

The 126-fold difference in nDNA content between the two fractions confirms that selective enrichment of mtDNA was obtained through differential centrifugation and PCI extraction. Previously, comparable nDNA: mtDNA ratios for the mitochondrial and nuclear fraction were obtained using silica-coated columns^24^. Whilst this DNA extraction technique is fast and does not involve toxic chemicals, silica nanoparticles can trigger complement activation, which limits their utility given our downstream application^25^. Additionally, mtDNA yields are lower with silica-columns compared to PCI which could be caused by the tight binding of short DNA fragments to the column and the inability of the method to recover these fragments^26^.

Most human studies that isolated mtDNA for experimental purposes either extracted a single DNA fraction or employed different methods to isolate both fractions, thereby accepting the risk of yielding a false-positive result as the fractions may include different contaminants^6,27,28^. We ought to overcome these issues by isolating nDNA and mtDNA fractions in parallel from the same tissue and treated both with identical reagents. In addition, we included a cell-free PBS sample, which was subjected to the same extraction procedure, to rule-out the impact of potential contaminations on complement activation.

Extracellular mitochondria has been shown to activate the complement system, which was mediated by the lectin pathway upon recognition by MBL, L-ficolin, and M-ficolin leaded to C3 consumption in a mouse model^29^. Recently, we reported that synthetic CpG-ODNs, which mimic the immunostimulatory activity of bacterial and mtDNA, directly activated the complement system in whole blood and plasma through the classical and lectin pathways^11^. In the current study, we confirm that endogenous mtDNA activated the complement system at a level comparable to the synthetic CpG-ODNs.

The relation between extracellular mtDNA and complement activation in sterile inflammation is still unexplored. Here, we show that extracellular mtDNA and nDNA released following OHCA, strongly correlates with complement activation. Previously, DAMP properties of extracellular mtDNA were shown by mtDNA inducing activation of p38 MAP kinase, nuclear factor kappa B and myocardial cell death^30,31^. Our results provide further evidence that the excessive release or active secretion of mtDNA by injured cells lead to a pro-inflammatory phenotype observed in OHCA patients, possibly mediated through complement activation^32^. The mtDNA concentrations measured in OHCA patient samples collected 1–2 h(s) after ROSC are rather low, which might be explained by the rapid breakdown of mtDNA in humans^33^. In vitro, we did not observe any activation of the complement system with nDNA. However, we observed a noteworthy association between nDNA and complement levels in OHCA patients. nDNA has previously been described as a marker for cellular damage, which is a hallmark in OHCA patients^34–36^. Moreover, we found a strong correlation between mtDNA and nDNA in our patient population. This might imply that the observed association between nDNA and complement activation in vivo could also be linked to the release of mtDNA as both nDNA and mtDNA are simultaneously released upon cell injury.

Whether or not extracellular mtDNA relates to endothelial damage in OHCA patients remains unclear. In contrast to previous research^37^, none of the endothelial markers were correlated to the level of mtDNA. However, endothelial cell activation is not solely dependent on complement activation, but rather a result of a complex inflammatory reaction induced over days involving multiple types of blood cells, especially neutrophils. While extracellular mtDNA may activate neutrophils, which can lead to the release of mtDNA^31,38^, only a sustained release of mtDNA by activated neutrophils has been shown to trigger TM and ICAM-1 expression in patients undergoing cardiopulmonary bypass > 100 min^37^. While cardiac bypass induced both complement and neutrophil activation directly through biomaterial interaction^39,40^, cardiac arrest and resuscitation lead to inflammation through hypoxic induced tissue injury, resulting in larger inter-patient variations. Thus, future studies should evaluate if mtDNA can directly stimulate endothelial cell activation in vitro or if an interplay of mtDNA with the innate immune system is a prerequisite for tissue inflammation.

In infection-driven inflammation, a positive correlation was reported between the level of circulating mtDNA and sC5b-9^15^. Recognition that immune responses may appear similar in sterile and infectious conditions is of critical importance in the search for novel treatment strategies. The blockade of either C5 or TLR-9, activated in a CD14-dependent manner, has already been shown to reduce cardiac IRI^41,42^. Given that both the complement system and TLR-9 act as upstream sensors of extracellular mtDNA, combined inhibition of complement and TLRs, through e.g. C5 and CD14 inhibition, might be a novel therapeutic strategy to attenuate sterile inflammation^43^. However, further research will be required to assess this concept.

The strengths of this study are manifold. First, we evaluate the impact of mtDNA on complement activation at physiologically relevant mtDNA concentrations. Previous research found that mtDNA concentrations ranged from 5,279 to 8,586 copies/µl in plasma samples collected at admission from trauma patients^44,45^. Moreover, Nakahira et al. reported that medical ICU patients with mtDNA concentrations above 3,200 copies/µl had an increased risk of death^46^. In the current study, we incubated 300–9,000 copies/µl of mtDNA in either whole blood or plasma, which covered a wide range of mtDNA concentrations reported previously (Fig. 2). Second, we used an ex vivo human whole-blood model anticoagulated with lepirudin. Since lepirudin does not exert any known influence on the complement cascade, the whole blood model used in this study is, to the best of our knowledge, one of the most physiologically relevant whole blood models^18,47^.

One limitation of this study is the limited availability of samples, which precluded additional pathway-specific inhibition experiments to conclusively pinpoint the mechanism of mtDNA driven complement activation. Based on our previous findings, we hypothesizes that activation involves both classical and alternative pathways, possibly mediated through the mtDNA backbone, as supported by the observed increase in pathway-dependent C3bc formation in whole blood and plasma^11^; further research is needed to confirm this hypothesis. Another limitation is that DNase pre-treatment could not be performed due to the limited availability of mtDNA material, although DNA-free controls were included. Lastly, no statistically significant associations of mtDNA content and clinical outcome variables were found. However, the NORCAST sub study was limited by small sample size and limited clinical data, warranting validation in larger cohorts with comprehensive outcomes.

## Conclusion

In conclusion, we show for the first time that endogenous mtDNA activated the complement system in a dose- and time-dependent manner in human plasma and a whole blood model. Further, we observed a robust correlation between mtDNA levels and the extent of complement activation in OHCA patients. While this finding suggests a potential physiological link, confirmation of such a process requires evidence from in-vivo or ex-vivo models. The recognition of mtDNA’s potent ability to trigger the complement system, while nDNA appears to have minimal impact in this context, provides mechanistic evidence for the activation of the immune system in response to cellular damage, unveiling potential avenues for novel therapeutical interventions during sterile inflammatory processes.

## Supplementary Information

Below is the link to the electronic supplementary material.

Supplementary Material 1

## Data Availability

The datasets generated during and/or analysed during the current study are available from the corresponding author on reasonable request.
