# Supplementary material for: Extracellular mitochondrial DNA activates complement and is associated with complement activation in patients with out-of-hospital cardiac arrest
Source: Sci Rep. 2025 Nov 20;15:41000. doi: 10.1038/s41598-025-24705-1 (PMC12635304; doi:10.1038/s41598-025-24705-1)
Supplement: Supplementary file 1 — Supplementary Material 1 [file 41598_2025_24705_MOESM1_ESM.pdf]

Extracellular mitochondrial DNA activates complement and is associated with complement activation in patients with out-of-hospital cardiac arrest

Eline de Boer, Anne-Lise Strandmoe, Marina Sokolova, Trond M. Michelsen, Huy Q. Quach, Viktoriia Chaban, Espen R. Nakstad, Geir Ø. Andersen, May-Kristin S. Torp, Kåre-Olav Stensløkken, Tom E. Mollnes, Søren E. Pischke

Supplemental Table 1. Results of correlation analysis and linear regression model assessing association between mtDNA, complement variates and clinical outcome (n=55).

	Correlation analysis		Regression analysis	
	r	p-value	B (95% CI)	p-value
mtDNA, SOFA score	0.293	**0.037**	0.000 (-0.002-0.001)	0.771
mtDNA, creatinine levels	0.441	**0.001**	0.000 (0.000-0.000)	0.602
mtDNA, Time-to-ROSC	0.321	**0.036**	0.002 (-0.009-0.013)	0.711
mtDNA, neurological outcome (CPC score)	0.160	0.242	0.000 (-0.001-0.001)	0.935
sC5b-9, SOFA score	0.282	**0.045**	0.185 (-0.002-0.001)	**0.049**
sC5b-9, creatinine levels	0.388	**0.005**	0.019 (-0.021-0.059)	0.340
sC5b-9, Time-to-ROSC	0.250	0.105	0.038 (-0.033-0.109)	0.287
sC5b-9, neurological outcome (CPC score)	0.043	0.762	0.038 (-0.135-0.210)	0.662
C3bc, SOFA score	0.129	0.365	0.027 (-0.005-0.049)	**0.017**
C3bc, creatinine levels	0.267	0.056	0.003 (-0.002-0.008)	0.227
C3bc, Time-to-ROSC	0.271	0.078	0.005 (-0.004-0.014)	0.277
C3bc, neurological outcome (CPC score)	0.000	0.998	0.001 (-0.020-0.022)	0.947

Correlation, Spearman rank correlation analysis; Regression, linear regression analysis on (ln-transformed) values; r, correlation coefficient; CI, confidence interval; mtDNA, mitochondrial DNA; sC5b-9, soluble C5b-9; SOFA, sequential organ failure assessment; ROSC, return of spontaneous circulation; CPC, cerebral performance category.
